# Renal Hemodynamic and Morphological Changes after 7 and 28 Days of Leptin Treatment: The Participation of Angiotensin II via the AT_1_ Receptor

**DOI:** 10.1371/journal.pone.0122265

**Published:** 2015-03-20

**Authors:** Karina Thieme, Maria Oliveira-Souza

**Affiliations:** Department of Physiology and Biophysics, Institute of Biomedical Sciences, University of São Paulo, São Paulo, Brazil; INSERM, FRANCE

## Abstract

The role of hyperleptinemia in cardiovascular diseases is well known; however, in the renal tissue, the exact site of leptin’s action has not been established. This study was conducted to assess the effect of leptin treatment for 7 and 28 days on renal function and morphology and the participation of angiotensin II (Ang II), through its AT_1_ receptor. Rats were divided into four groups: sham, losartan (10 mg/kg/day, s.c.), leptin (0.5 mg/kg/day for the 7 days group and 0.25 mg/kg/day for the 28 days group) and leptin plus losartan. Plasma leptin, Ang II and endothelin 1 (ET-1) levels were measured using an enzymatic immuno assay. The systolic blood pressure (SBP) was evaluated using the tail-cuff method. The renal plasma flow (RPF) and the glomerular filtration rate (GFR) were determined by p-aminohippuric acid and inulin clearance, respectively. Urinary Na^+^ and K^+^ levels were also analyzed. Renal morphological analyses, desmin and ED-1 immunostaining were performed. Proteinuria was analyzed by silver staining. mRNA expression of renin-angiotensin system (RAS) components, TNF-α and collagen type III was analyzed by quantitative PCR. Our results showed that leptin treatment increased Ang II plasma levels and progressively increased the SBP, achieving a pre-hypertension state. Rats treated with leptin 7 days showed a normal RPF and GFR, but increased filtration fraction (FF) and natriuresis. However, rats treated with leptin for 28 showed a decrease in the RPF, an increase in the FF and no changes in the GFR or tubular function. Leptin treatment-induced renal injury was demonstrated by: glomerular hypertrophy, increased desmin staining, macrophage infiltration in the renal tissue, TNF-α and collagen type III mRNA expression and proteinuria. In conclusion, our study demonstrated the progressive renal morphological changes in experimental hyperleptinemia and the interaction between leptin and the RAS on these effects.

## Introduction

Leptin is a small 16 kDa peptide secreted by adipose tissue that, in physiological conditions, feeds back to inform the central nervous system about the status of peripheral energy reserves, thereby regulating appetite and energy expenditure [1, 2]. The knowledge about its biological actions increased substantially over the last decades and it is now known that leptin also exerts an important role on sympathetic nerve activity (SNA), immune function, cardiovascular and renal systems [3].

The biological action of leptin depends on its interaction with a family of class I cytokine receptors identified as Ob-Ra to Ob-Rf [4]. The full-length leptin receptor, Ob-Rb, is highly expressed in the hypothalamus; however, its expression has been demonstrated in other tissues, including blood vessels [5] and the kidneys [6].

In the kidneys, leptin is filtered and then taken up by the megalin receptor in the proximal convolute tubule cells [7] and almost no leptin is found in the urine [8]. Apart from its processing, leptin has direct and indirect effects on renal pathophysiology. In the renal tissue, the exact site of leptin’s action has not been established. However, the identification of the short isoform of the leptin receptor (Ob-Ra) in the glomerular endothelial and mesangial cells [9, 10] and the expression of the long isoform (Ob-Rb) in the renal medulla, suggests that the glomerulus and the collecting ducts are important target sites for leptin’s direct action [11]. In addition, studies have previously demonstrated that leptin increases the expression of transforming growth factor-β1 (TGF-β1) and its receptor (TβRII); the synthesis of collagen type I in mesangial cells; and induces proliferation of glomerular endothelial cells [4, 9, 10]. Other studies demonstrated that long-term leptin treatment exerts a pro-apoptotic effect on renal tubular cells, confirming that this peptide is an important component in the development of kidney diseases [12, 13]. Nonetheless, leptin’s chronic effect remains controversial and depends on the dose, time and administration route [14–18]. In addition, the indirect and long-term effects of leptin on renal hemodynamic, glomerular function and morphology remains unclear.

High-fat diet-induced obesity or chronic leptin infusion are correlated with increased arterial blood pressure [14, 19]. The mechanisms by which hyperleptinemia contributes to hypertension include the following: activation of the sympathetic nervous system innervating the kidneys [20, 21], increase in circulating endothelin-1 (ET-1) [22], increase in oxidative stress, decrease in nitric oxide [15, 16] and increase in sodium retention [16, 23].

It is known that the increased SNA to the kidneys can also activate the renin-angiotensin system (RAS), whose major effector is the octapeptide angiotensin II (Ang II). Ang II is a multifunctional hormone that regulates physiological processes such as blood pressure, plasma volume, renal hemodynamic and excretory functions. All of these actions result from the binding of Ang II to one of its membrane receptors, AT_1_ or AT_2_ [24]. The interaction between Ang II and the AT_1_ receptor is particularly important in renal injury because it increases the intraglomerular pressure, induces inflammation, cellular growth, apoptosis, cellular migration and differentiation [25]. In addition, several studies have demonstrated that the RAS components are synthesized in many tissues, including the heart and the kidneys [26, 27].

Considering that chronic leptin treatment mimics the hyperleptinemic state without the influence of other obesity-associated factors [17, 28–33] and that leptin also acts indirectly on the renal tissue, we hypothesized that leptin infusion could induce RAS activation and consequently, changes in blood pressure, renal function and morphology. Thus, we treated rats for 7 and 28 days and evaluated the effects of leptin on body weight, systolic blood pressure, renal function and morphology and intrarenal mRNA expression of RAS components, inflammatory and fibrotic factors. We also investigated the contribution of Ang II/AT_1_ receptor, in the leptin’s effects.

## Materials and Methods

### Ethics statement

All of the experimental protocols were conducted in accordance with the guidelines of the Brazilian College for Animal Experimentation (COBEA) and were approved by the Ethical Committee for Animal Research of the Institute of Biomedical Sciences of the University of São Paulo (Protocol Number 150, page 78, book 2). Experiments were performed with 50 male Wistar rats weighing between 150 and 250 g (mean: 206.2 ± 3.20 g). The rats were obtained from colonies and maintained at the Animal Quarters of the Department of Physiology and Biophysics of the Institute of Biomedical Science, University of São Paulo. The rats were housed under standard conditions (constant temperature of 22°C, 12-h dark-light cycle, and relative humidity of 60%) with free access to standard rat chow and tap water.

### Animals

The rats were randomly assigned to one of the following groups and were treated for 7 or 28 days. The 7-day treatment groups included: sham (saline; n = 6); losartan (10 mg/kg/day; n = 6) [18] (Tocris Bioscience, Bristol, United Kingdom); leptin (recombinant rat leptin; 0.5 mg/kg/day; n = 6) [16] (R&D Systems, Minneapolis, USA); and leptin plus losartan (n = 6). The 28-day treatment groups included: sham (n = 6); losartan (n = 6); leptin (0.25 mg/kg/day; n = 8); and leptin plus losartan (n = 6). The rats were anesthetized using Francotar (ketamine 75 mg/kg, i.p.; Virbac, Carros, France) and Virbaxyl 2% (xylazine, 4 mg/kg, i.p.; Virbac). Osmotic mini-pumps (2ML1 or 2ML4 Alzet, Cupertino, USA) were implanted in the midscapular region for continuous infusion of leptin or saline for 7 or 28 days, respectively. Losartan was administered daily via subcutaneous injection, for the same period of time as the leptin treatment.

### Food intake and body weight gain

After osmotic mini-pump implantation, the animals were individually housed in normal cages and the food intake and body weight (BW) were evaluated. Food intake is an average *per day* of the total food intake during the treatment period and body weight gain was calculated using the following equation: (initial BW—final BW)/initial BW.

### Systolic blood pressure (SBP)

The SBP was recorded before the start of the experiment and on the last day of treatment in the 7-day groups and weekly in the 28-day groups. The SBP was measured in conscious, resting animals using noninvasive tail-cuff plethysmography (LE, 5001 Non Invasive Blood Pressure Meter, Panlab/Harvard Apparatus, Barcelona, Spain). The rats were acclimatized to the apparatus during daily sessions, before the initial measurement, to assure accurate measurements. The tail artery was dilated by placing the animal into a thermostatically controlled plastic holder that was heated for 20 minutes. The pulse was detected by passing the tail through a tail-cuff sensor that was attached to the amplifier. Systolic blood pressure measurements were considered to be successful if the rat did not move and a clear initial and constant pulse could be detected. The average values for the SBP were subsequently obtained from eight sequential cuff inflation-deflation cycles.

### Plasma peptide levels

The plasma levels of leptin, Ang II and ET-1 were measured by enzymatic immuno assay (EIA) using specific kits for each protein (Phoenix Pharmaceuticals Inc., Burlingame, USA), according to the manufacturer’s instructions.

### Renal function studies

After 7 or 28 days of treatment, the rats were anesthetized with Zoletil (zolazepam, 50 mg/kg; Virbac) and Virbaxyl 2% (xylazine, 5 mg/kg) and placed on a warm table to maintain the body temperature at 37°C. Supplemental doses of the anesthetic were administered as required. A tracheotomy using PE-260 tubing was performed, the right carotid artery was catheterized with PE-50 tubing for blood sampling, and the right jugular vein was catheterized with PE-50 tubing for continuous fluid infusion. The bladder was catheterized with PE-260 via a suprapubic incision to allow timed urine collections. After a 60-minute equilibration period, four consecutive 30-minute urine and arterial blood samples (500 μL) were obtained to determine renal hemodynamic and excretory parameters. The animals were first primed with 1 mL of a solution containing inulin (300 mg/kg; Sigma Aldrich, St Louis, USA) and sodium *para*-aminohippuric acid (PAH, 2 mg/rat; Sigma Aldrich). Subsequently, the rats were given a continuous infusion of 0.9% NaCl plus 3% mannitol containing inulin (15 mg/mL) and PAH (4 mg/L) at 0.1 mL/min using an infusion pump (Harvard Instruments, Holliston, USA). Plasma and urine inulin and PAH concentrations were measured by standard colorimetric techniques using a spectrophotometer (μQuant, Bio-Tek Instruments Inc., Winooski, USA) within 24 hours to avoid sample degradation. The glomerular filtration rate (GFR) was calculated from inulin clearance [34], and the PAH clearance [35] was used as an index of the renal plasma flow (RPF). The blood samples were centrifuged at 1,500 g and 4°C to obtain plasma. The urine volume for each collection (total of 4 collections) was measured and used to calculate the urinary flow rate, using the following formula: urine volume/time (mL/min). The RPF and the GFR were normalized by body weight and are shown as mL/min/kg. The FF % was calculated using the following equation: GFR/RPF × 100.

### Urine and plasma parameters

The sodium and potassium concentrations in urine and plasma were determined by flame photometry (9180 Electrolyte Analyzer, Roche, Mt. Wellington, Auckland, New Zealand). The fractional ion excretion (FE%) was calculated using the formula: (V × [X^+^]_u_/[X^+^]_p_ × GFR) × 100, where V is the urinary flow rate, [X^+^]_u_ is the urine sodium or potassium concentration, and [X^+^]_p_ is the plasma sodium or potassium concentration.

### Morphological and immunohistochemical studies

One kidney was used for quantitative PCR experiments and the other one was fixed in 4% paraformaldehyde for histological studies. Four micrometer histological sections were stained with hematoxylin and eosin (HE) and examined under a light microscope (Eclipse 80*i*, Nikon, Tokyo, Japan). To analyze the planar glomerular area, the outer edges of 50 glomerular tufts *per* animal were traced on a video screen and the encircled areas were analyzed by a computerized morphometry program (NIS-Elements D, Nikon). To measure the fractional interstitial area (FIA) from renal cortex, 15 grid fields (167118.9 μm^2^) *per* animal were analyzed and the interstitial areas were traced manually on a video screen. The fraction occupied by the interstitium was then determined. Additionally, the kidney sections were subjected to immunohistochemical staining for desmin (Abcam, Cambridge, UK) and ED-1 (AbD Serotec, Oxford, UK). The tissue sections were deparaffinized and nonspecific protein binding was blocked by incubation with 20% goat serum in PBS for 60 min [36] followed by incubation with primary antibodies (1: 500 for desmin and 1:50 for ED-1), overnight at 4°C. The reaction products were detected using the avidin-biotin-peroxidase complex (Vector Labs, Burlingame, USA) and the sections were counterstained with methyl green (Amresco, Ohio, USA), dehydrated and mounted with Permount (Fisher Scientific, Fair Lawn, USA). The mean number of ED1-positive cells (macrophages) infiltrating the renal cortical tubulointerstitium was obtained by evaluating 50–60 grid fields (measuring 0.087 mm^2^ each) and by calculating the mean counts *per* kidney [37].

### Albuminuria

The urinary proteins were analyzed using a ProteoSilver Plus Silver Stain Kit (Sigma Aldrich), according to the manufacturer’s instructions. This kit utilizes silver nitrate that binds to certain amino acids on the proteins under specific pH conditions. The urine samples were resolved on 10% SDS page gels, which were fixed overnight with an ethanol/water/glacial acetic acid mixture (50:40:10). The following day, the gels were exposed to a sensitizer solution, the silver was impregnated, and finally, the image was developed. The bands were analyzed by optical densitometry using the Scion Image software (Scion Corporation, Frederick, USA).

### Intrarenal mRNA expression

After the renal function studies, one kidney was removed and quickly frozen and pulverized in liquid nitrogen, followed by the isolation of RNA using the TRIzol LS Reagent (Life Technologies, Carlsbad, USA) and a RNA extraction kit (Qiagen Sciences, Germantown, USA). RNA was quantified measuring the optical density at 260 nm and was then stored at-80°C. Total RNA (2 μg) was reverse-transcribed using random hexamers (High Capacity cDNA Reverse Transcription Kit; Life Technologies, Carlsbad, USA) following the manufacturer's guidelines. Real-time PCR was performed using the StepOnePlus System (Life Technologies, Carlsbad, USA). To analyze the expression of the short (Ob-Ra) and the long (Ob-Rb) leptin receptors, SYBR green qPCR studies were performed, using the following primers: Ob-Ra (GI: 1526439) sense: 5′-GCTGCTCGGAACACTGTTAAT-3′ and antisense: 5′GAGTGTCCGCTCTCTTTTGG-3′; Ob-Rb (GI: 9587399) sense: 5′-CCTGCTGGAGTCCCAAACAA-3′ and antisense: 5′-GCGGAGCAGTTTTGACCTTG-3′. For the other genes, the expression analysis was performed using the TaqMan assay system (Life Technologies, Carlsbad, USA) and all assays probes spanned an exon junction. The following assays were used: angiotensinogen: Rn00593114_m1; renin: Rn00561847_m1; angiotensin converting enzyme (ACE): Rn00561094_m1; collagen type III (Col3a1): Rn01437681_m1; tumor necrosis factor (TNF-α): Rn99999017_m1; endothelin 1 (ET-1): Rn00561129_m1 and GAPDH: Rn01775763_g1 (internal control). All quantitative PCR studies were performed using 20 ng of cDNA and all samples were assayed in duplicate. The comparative cycle threshold method was used for data analysis. The data were normalized using GAPDH and were expressed as the fold change relative to the sham group.

### Statistical analysis

The data are reported as the mean ± SEM. For comparisons among the groups, one-way ANOVA followed by Bonferroni’s test (GraphPad Prism Software, San Diego, USA) was performed. The differences with *p* <0.05 were considered statistically significant.

## Results

### Food intake and body weight gain

As already demonstrated by other studies [18, 38] no differences in food intake in animals treated with leptin for 7 and 28 days were found; therefore, a *pair-fed* group was not necessary. However, despite similar food intake, the rats treated with leptin for 7 days showed less body weight gain and the rats treated with leptin plus losartan, showed a partial recovery of this parameter. Losartan alone did not change the body weight gain. No differences in body weight gain were observed in the rats treated for 28 days (Table 1).

**Table 1:** Food intake and body weight gain of rats treated for 7 and 28 days.

	Sham	Losartan	Leptin	Leptin plus Losartan
***7 days***	n = 6	n = 6	n = 6	n = 6
**Food intake (g/day)**	20.64 ± 0.89	20.00 ± 1.18	17.30 ± 1.14	19.81 ± 1.02
**BW gain (%)**	17.33 ± 1.90	16.15 ± 1.16	3.62 ± 1.17*	10.71 ± 1.88* ^#^
***28 days***	n = 6	n = 6	n = 8	n = 6
**Food intake (g/day)—week 1**	20.87 ± 3.46	23.57 ± 2.10	20.46 ± 2.36	19.19 ± 3.33
**Food intake (g/day)—week 2**	26.78 ± 2.00	26.68 ± 1.03	23.60 ± 2.65	23.48 ± 2.92
**Food intake (g/day)—week 3**	25.68 ± 2.33	25.89 ± 1.92	24.37 ± 2.82	24.12 ± 3.42
**Food intake (g/day)—week 4**	22.00 ± 0.83	20.97 ± 1.62	24.52 ± 3.30	25.66 ± 2.38
**Body weight gain (%)**	45.65 ± 0.83	44.35 ± 2.49	41.05 ± 2.62	39.23 ± 1.95

*p< 0.05 *versus* Sham;

^#^p< 0.05 *versus* Leptin

### Systolic Blood Pressure (SBP)

**Fig 1:**
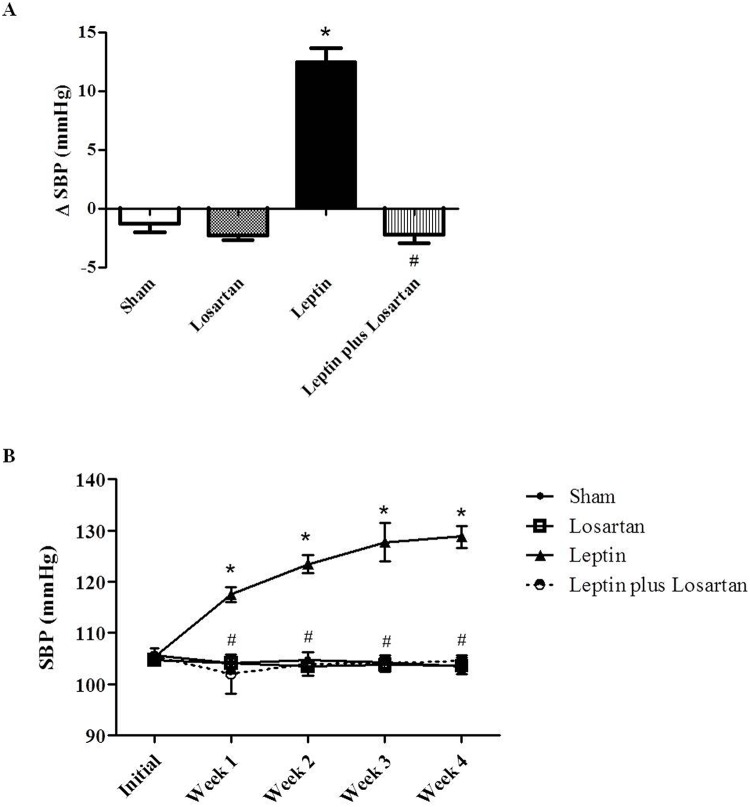
Systolic blood pressure. The delta of the systolic blood pressure (SBP, in mmHg) of rats treated for 7 days (A) and the SBP progression of the rats treated for 28 days (B). Values are presented as the mean ± SE for the representative groups. *p < 0.05 *versus* the sham and ^#^p < 0.05 *versus* the leptin-treated rats. For the rats treated for 7 days: sham (n = 6); losartan (n = 6); leptin (n = 6); and leptin plus losartan (n = 6). For the rats treated for 28 days: sham (n = 6); losartan (n = 6), leptin (n = 8); leptin plus losartan (n = 6).

Many studies suggest an important participation of leptin in obesity-related hypertension [20, 21]. In the present study, the time-course changes in SBP from rats treated for 7 and 28 days were analyzed. The initial SBP was similar in all of the groups, whereas the final SBP measurement was higher in the rats treated with leptin, from both treatment periods (Table 2). Losartan alone did not change the final SBP compared to the sham rats, but it prevented the elevation of the SBP in the rats treated with leptin from both treatment periods. The delta of the SBP of rats treated for 7 days is shown in Fig. 1A. The SBP over the 4 weeks in the leptin-treated rats for 28 days is exhibited in Fig. 1B.

**Table 2:** Systolic blood pressure (SBP, in mmHg) pressure and leptin levels of rats treated for 7 and 28 days.

	Sham	Losartan	Leptin	Leptin plus Losartan
***7 days***	n = 6	n = 6	n = 6	n = 6
**Initial SBP (mmHg)**	107.10 ± 0.50	108.00 ± 0.94	105.5 ± 0.62	105.6 ± 0.27
**Final SBP (mmHg)**	105.8 ± 0.66	105.6 ± 1.14	118.00 ± 1.53*	103.4 ± 0.70^#^
**Plasma leptin (pg/mL)**	1433 ± 191	1721 ± 159	4083 ± 634*	3518 ± 258*
***28 days***	n = 6	n = 6	n = 8	n = 6
**Initial SBP (mmHg)**	105.6 ± 1.39	105.3 ± 0.87	105.3 ± 0.42	105.43 ± 0.71
**SBP—week 1 (mmHg)**	104.1 ± 0.71	104.1 ± 0.45	117.5 ± 1.43*	101.2 ± 3.92^#^
**SBP—week 2 (mmHg)**	104.7 ± 0.59	103.5 ± 0.84	123.4 ± 1.76*	103.9 ± 2.31^#^
**SBP—week 3 (mmHg)**	104.2 ± 0.33	103.7 ± 1.37	127.7 ± 3.75*	104.0 ± 1.50^#^
**SBP—week 4 (mmHg)**	103.5 ± 1.07	103.6 ± 1.62	128.8 ± 2.14*	104.5 ± 1.16^#^
**Plasma leptin (pg/mL)**	1742 ± 601.5	1628 ± 149.3	7751 ± 417.8*	3928 ± 660.5^#^
**Plasma Ang II (ng/L)**	13.22 ± 2.18	28.83 ± 3.35	38.91 ± 3.83*	41.27 ± 9.29*
**Plasma ET-1 (ng/mL)**	36.25 ± 1.14	31.59 ± 2.25	34.24 ± 2.89	28.17 ± 1.16

*p< 0.05 *versus* Sham;

^#^p< 0.05 *versus* Leptin.

### Plasma peptide levels

As expected, plasma leptin levels were significantly elevated in the leptin-treated rats from both treatment periods (7 and 28 days) (Table 2). In the rats treated with leptin plus losartan for 7 days, the leptin plasma levels were also elevated; however, in the rats treated with leptin plus losartan for 28 days, there was a reduction in the plasma leptin level. Considering the progressive increase in the SBP observed in the group treated for 28 days, the plasma Ang II and ET-1 levels were analyzed only in this group. The results showed a significant increase in the plasma Ang II levels in both the leptin and leptin plus losartan groups. However, the plasma ET-1 levels were not changed with leptin and/or losartan treatment.

### Renal hemodynamics

**Fig 2:**
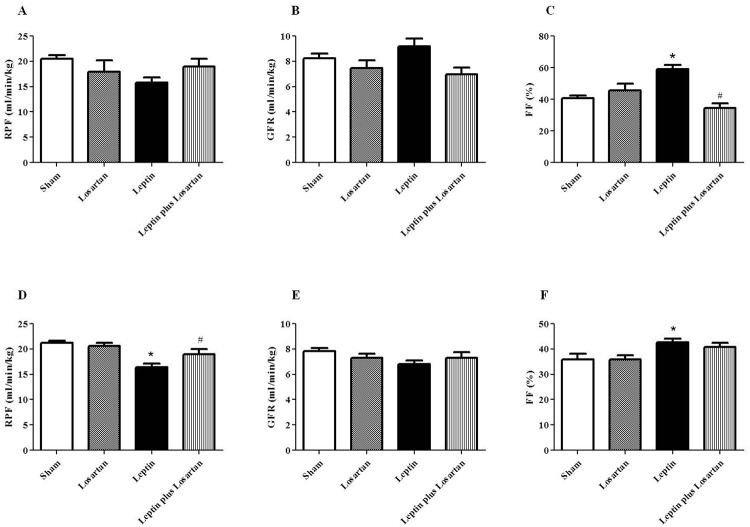
Renal hemodynamic. (A, D) The renal plasma flow (in mL/min/kg) of the rats treated for 7 and 28 days, respectively; (B, E) the glomerular filtration rate (in mL/min/kg) of the rats treated for 7 and 28, respectively; and (C, F) the filtration fraction (in %) of the rats treated for 7 and 28, respectively. *p<0.05 *versus* the sham and ^#^p<0.05 *versus* the leptin-treated rats. For the rats treated for 7 days: sham (n = 6); losartan (n = 6); leptin (n = 6); and leptin plus losartan (n = 6). For the rats treated for 28 days: sham (n = 6); losartan (n = 6), leptin (n = 8); leptin plus losartan (n = 6).

Because leptin increases sympathetic nerve activity to the kidneys, which could secondarily activate the RAS (an important regulator of renal hemodynamics), we next examined if leptin could induce changes in renal hemodynamic parameters and if Ang II, via the AT_1_ receptor participates in these effects. In the rats treated for 7 days, the renal plasma flow (RPF) was not significantly different among the groups, but leptin treatment decreased the RPF by 23%, compared with the sham group (in mL/min/kg: sham = 20.48 ± 0.77; losartan = 17.96 ± 2.21; leptin = 15.79 ± 1.02 and leptin plus losartan = 18.99 ± 1.54) (Fig. 2A). In contrast, in the rats treated for 28 days, the leptin group showed a significant decrease in RPF, which was normalized in leptin plus losartan group (in mL/min/kg: sham = 21.22 ± 0.46; losartan = 20.58 ± 0.61; leptin = 16.44 ± 0.71* and leptin plus losartan = 19.00 ± 0.99^#^, *p<0.05 *versus* the sham group and ^#^p<0.05 *versus* the leptin-treated rats) (Fig. 2D). The glomerular filtration rate (GFR) was also not significantly different among the groups treated for 7 days, with only a slight increase (12%) in the leptin-treated rats compared with the sham group (in mL/min/kg: sham = 8.24 ± 0.36; losartan = 7.46 ± 0.61; leptin = 9.19 ± 0.59 and leptin plus losartan = 6.98 ± 0.52) (Fig. 2B). Likewise, the GFR was also not different among the groups treated for 28 days (in mL/min/kg: sham = 7.83 ± 0.24; losartan = 7.31 ± 0.32; leptin = 6.79 ± 0.32 and leptin plus losartan = 7.31 ± 0.45) (Fig. 2E). The rats treated with leptin for 7 days showed a decrease in the RPF and a slight increase in the GFR. Thus, the filtration fraction (FF) increased significantly in this group and was normalized in the leptin plus losartan-treated group (in %: sham = 40.59 ± 1.83; losartan = 45.54 ± 4.27; leptin = 59.09 ± 2.46* and leptin plus losartan = 34.42 ± 3.06^#^; *p<0.05 *versus* the sham group and ^#^p<0.05 *versus* the leptin-treated rats) (Fig. 2C). The rats treated with leptin for 28 days also showed an enhanced FF, which was normalized in the leptin plus losartan group (in %: sham = 35.95 ± 2.27; losartan = 35.87 ± 1.57; leptin = 42.70 ± 1.36* and leptin plus losartan = 40.88 ± 1.58^#^; *p<0.05 *versus* the sham group and ^#^p<0.05 *versus* the leptin-treated rats) (Fig. 2F).

### Tubular function

**Fig 3:**
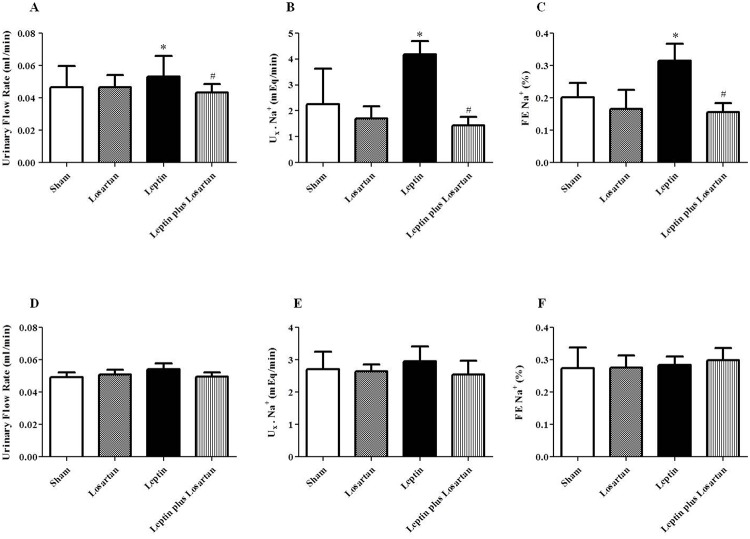
Tubular function. (A, D) The urinary flow rate (in mL/min) of the rats treated for 7 and 28 days, respectively; (B, E), urinary Na^+^ excretion (in mEq/min) of the rats treated for 7 and 28 days, respectively; (C, F) fractional excretion of Na^+^ (in %) of the rats treated for 7 and 28 days, respectively^. *^p<0.05 *versus* the sham and ^#^p<0.05 *versus* the leptin-treated rats. For the rats treated for 7 days: sham (n = 6); losartan (n = 6); leptin (n = 6); and leptin plus losartan (n = 6). For the rats treated for 28 days: sham (n = 6); losartan (n = 6), leptin (n = 8); leptin plus losartan (n = 6).

Parallel to the normal GFR, the urinary flow rate was only enhanced in the rats treated with leptin for 7 days (7-day group in mL/min: sham = 0.047 ± 0.002; losartan = 0.047 ± 0.001; leptin = 0.53 ± 0.003* and leptin plus losartan = 0.043± 0.001^#^; 28-day group in mL/min: sham = 0.049 ± 0.003; losartan = 0.050 ± 0.003; leptin = 0.54 ± 0.004 and leptin plus losartan = 0.049± 0.002) (Table 3 and Fig. 3A-D). Concomitantly, the filtered load of Na^+^ did not differ among the groups. The excretion and fractional excretion of Na^+^ was significantly increased only in the rats treated with leptin for 7 days and returned to control levels in the leptin plus losartan group (Table 3 and Fig. 3B-F). The filtered load, excretion (Table 3) and fractional excretion of K^+^ did not differ among the groups treated for 7 or 28 days.

**Table 3:** Tubular function of rats treated for 7 and 28 days.

	Sham	Losartan	Leptin	Leptin plus Losartan
***7 days***	n = 6	n = 6	n = 6	n = 6
**V (mL/min)**	0.047 ± 0.003	0.047 ± 0.002	0.053 ± 0.003*	0.043 ± 0.001^#^
**Plasma Na** ^**+**^ **(mEq/L)**	145.30 ± 1.34	145.40 ± 1.33	142.80 ± 1.69	143.8 ± 1.16
**Filtered Na** ^**+**^ **(mEq/min)**	1337 ± 129	1263 ± 180	1283 ± 108	914 ± 118
**U.Na** ^**+**^ **(mEq/min)**	2.26 ± 0.61	1.70 ± 0.23	4.17 ± 0.26*	1.44 ± 0.16^#^
**FE Na** ^**+**^ **(%)**	0.20 ± 0.02	0.17 ± 0.03	0.31 ± 0.03*	0.15 ± 0.01^#^
**Plasma K** ^**+**^ **(mEq/L)**	3.57 ± 0.30	3.20 ± 020	3.60 ± 0.25	3.20 ± 0.20
**Filtered K** ^**+**^ **(mEq/min)**	26.11 ± 4.53	21.72 ± 4.20	31.56 ± 3.99	19.00 ± 2.56
**U.K** ^**+**^ **(mEq/min)**	2.33 ± 0.31	1.89 ± 0.34	1.63 ± 0.14	1.70 ± 0.18
**FE K** ^**+**^ **(%)**	7.37 ± 1.18	8.00 ± 0.56	6.51 ± 0.88	8.58 ± 0.64
***28 days***	n = 6	n = 6	n = 8	n = 6
**V (mL/min)**	0.049 ± 0.003	0.051 ± 0.003	0.054 ± 0.004	0.050 ± 0.002
**Plasma Na** ^**+**^ **(mEq/L)**	143.20 ± 1.96	145.00 ± 1.32	143.80 ± 1.63	143.7 ± 1.73
**Filtered Na** ^**+**^ **(mEq/min)**	1052 ± 75	1010 ± 93	1028 ± 108	843 ± 54
**U.Na** ^**+**^ **(mEq/min)**	2.71 ± 0.53	2.63 ± 0.22	2.96 ± 0.45	2.54 ± 0.43
**FE Na** ^**+**^ **(%)**	0.27 ± 0.06	0.27 ± 0.04	0.28 ± 0.03	0.30 ± 0.04
**Plasma K** ^**+**^ **(mEq/L)**	3.80 ± 0.38	3.67 ± 0.33	3.20 ± 0.20	3.00 ± 0.00
**Filtered K** ^**+**^ **(mEq/min)**	27.26 ± 1.68	27.81 ± 3.68	25.67 ± 2.36	20.19 ± 1.32
**U.K** ^**+**^ **(mEq/min)**	1.63 ± 0.23	2.00 ± 0.21	2.79 ± 0.49	1.58 ± 0.24
**FE K** ^**+**^ **(%)**	6.11 ± 0.98	7.54 ± 0.82	10.08 ± 0.99	7.74 ± 1.08

V (urinary flow rate); U.Na^+^ (Na^+^ excretion); FE Na^+^ (fractional excretion of Na^+^); U.K^+^ (K^+^ excretion); FE K^+^ (fractional excretion of K^+^).

*p< 0.05 *versus* Sham;

^#^p< 0.05 *versus* Leptin.

### Renal morphology

**Fig 4:**
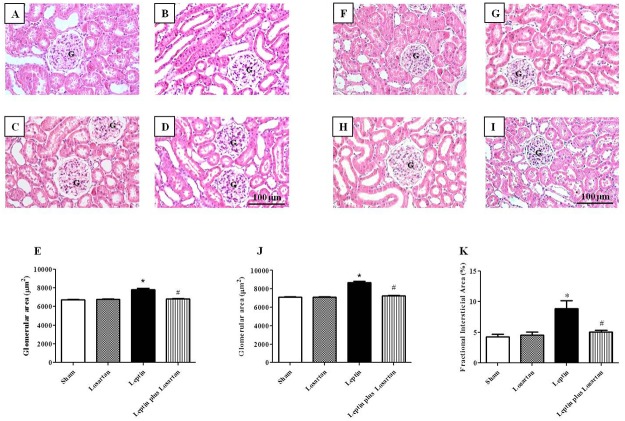
Renal morphology. The renal morphology of the leptin and/or losartan-treated rats. A representative image of the glomeruli of the sham (A), losartan (B), leptin (C) and leptin plus losartan-treated rats (D) (×20). Statistical analysis of the glomerular area of the rats treated for 7 days (E). A representative image of the glomeruli of the sham (F), losartan (G), leptin (H) and leptin plus losartan-treated rats (I) (×20). Statistical analysis of the glomerular area of the rats treated for 28 days (J). Statistical analysis of the fractional interstitial area of the rats treated for 28 days (K). *p<0.05 *versus* the sham and ^#^p<0.05 *versus* the leptin-treated rats; G = glomeruli; bar = 100 μm. For the rats treated for 7 days: sham (n = 6); losartan (n = 6); leptin (n = 6); and leptin plus losartan (n = 6). For the rats treated for 28 days: sham (n = 6); losartan (n = 6), leptin (n = 8); leptin plus losartan (n = 6).

A significant and progressive increase in the glomerular area was observed in the rats treated with leptin for 7 and 28 days, which was normalized in the leptin plus losartan groups (7-day treatment, in μm^2^: sham = 6708 ± 62; losartan = 6779 ± 23; leptin = 7796 ± 130* and leptin plus losartan = 6822 ± 20^#^; 28-day treatment, in μm^2^: sham = 7090 ± 72; losartan = 7093 ± 39; leptin = 8689 ± 149* and leptin plus losartan = 7243 ± 74^#^; *p<0.05 *versus* the sham group and ^#^p<0.05 *versus* the leptin-treated rats (Fig. 4A-J). Morphological analysis of the renal cortex by HE staining also revealed an increase in the fractional interstitial area (FIA) of the rats treated with leptin for 28 days and a reduction in the leptin plus losartan group (in %: sham = 4.28 ± 0.43; losartan = 4.56 ± 0.49; leptin = 8.85 ± 1.34* and leptin plus losartan = 5.04 ± 0.33^#^; *p<0.05 *versus* the sham group and ^#^p<0.05 *versus* the leptin-treated rats; Fig. 4K).

### Immunohistochemical studies

**Fig 5:**
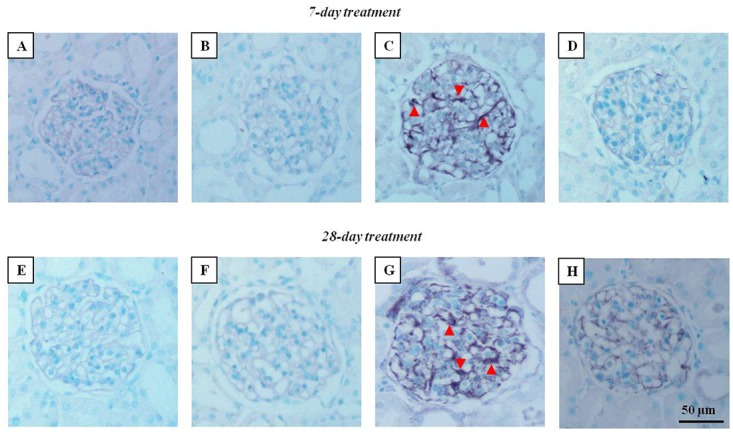
Desmin staining. Renal desmin immunostaining of the rats treated for 7 (A-D) and 28 days (E-H), ×20. Arrowheads = desmin staining; bar = 50 μm. For the rats treated for 7 days: sham (n = 6); losartan (n = 6); leptin (n = 6); and leptin plus losartan (n = 6). For the rats treated for 28 days: sham (n = 6); losartan (n = 6), leptin (n = 8); leptin plus losartan (n = 6).

**Fig 6:**
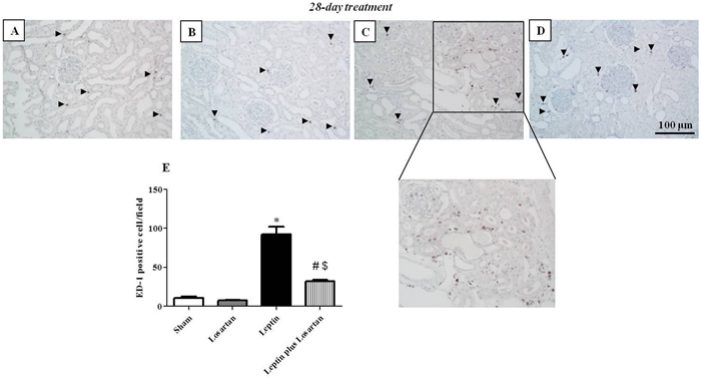
ED-1 staining. Renal ED-1 immunostaining of the rats treated for 28 days: sham (A), losartan (B), leptin (C) and leptin plus losartan (D). ED-1-positive cell quantification (in cells/area) (E). Magnification of the leptin-treated rats ED-1 positive cell area. G = glomeruli; arrowheads = ED-1-positive cells; bar = 100 μm. *p<0.05 *versus* the sham; ^#^p<0.05 *versus* the leptin-treated rats and ^$^p<0.05 *versus* the losartan-treated. For the rats treated for 28 days: sham (n = 6); losartan (n = 6), leptin (n = 8); leptin plus losartan (n = 6).

Although Gunduz *et al*.[18] showed an increase in TGF-β staining in the rats treated with leptin for 28 days, no studies have demonstrated glomerular injury in experimental hyperleptinemia. Our results showed that, along with glomerular hypertrophy, the rats treated with leptin for 7 and 28 days qualitatively exhibited increased desmin expression, as indicated by the arrowheads in Fig. 5A-H. In the leptin plus losartan group, the staining was decreased, indicating the contribution of Ang II, via the AT_1_ receptors, in the glomerular injury. Because the effects of leptin were more pronounced in the rats treated for 28 days, we also performed additional immunohistochemical studies for ED-1 (CD68). The anti-CD68 antibody used reacts to a cytoplasmic antigen present both in monocytes and macrophages [39]. Thus, ED-1 staining is a good marker of macrophage infiltration in the tissue. Our results showed a significant increase in this cell type in the cortical tubulointerstitium from the rats treated with leptin (in positive cells/field: sham = 10.75 ± 1.38; losartan = 7.5 ± 0,64; leptin = 92.5 ± 9.39* and leptin plus losartan = 31.75 ± 2.17^#$^; *p<0.05 *versus* the sham group; ^#^p<0.05 *versus* the leptin-treated rats and ^$^p<0.05 *versus* the losartan group) (Fig. 6A-D).

### Albuminuria

**Fig 7:**
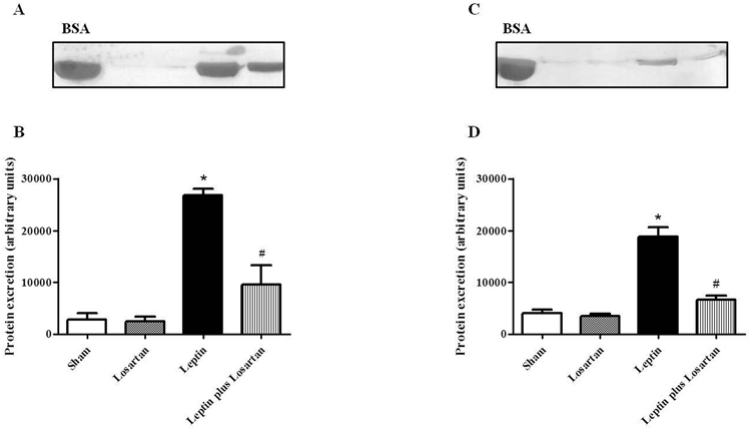
Albuminuria. Albuminuria of leptin and/or losartan-treated rats. Urine samples of rats treated for 7 (A) and 28 days (C) were resolved in 10% SDS page gels and stained using a ProteoSilver Plus Silver Stain Kit, according to the manufacturer’s instructions. The leptin-treated rats exhibited marked proteinuria in contrast to the sham group and the losartan-treated rats, whereas the leptin plus losartan group showed an attenuated protein excretion. Statistical analysis of protein excretion of both treatment periods (B, D). *p<0.05 *versus* the sham and ^#^p<0.05 *versus* the leptin-treated rats. BSA = 69 kDa. For the rats treated for 7 days: sham (n = 6); losartan (n = 6); leptin (n = 6); and leptin plus losartan (n = 6). For the rats treated for 28 days: sham (n = 6); losartan (n = 6), leptin (n = 8); leptin plus losartan (n = 6).

Urine samples from rats treated with leptin for 7 and 28 days, but not from sham or losartan-treated rats, showed a marked increase in the silver staining of proteins, which was significantly decreased in the leptin plus losartan group (Fig. 7A-D) (7-day treatment, in arbitrary units: sham = 2819 ± 1229; losartan = 2498 ± 926; leptin = 26728 ± 1279* and leptin plus losartan = 9623 ± 3629^#^; 28-day treatment, in arbitrary units: sham = 4083 ± 595; losartan = 3479 ± 418; leptin = 18891 ± 1758* and leptin plus losartan = 6686 ± 743 ^#^; *p<0.05 *versus* the sham group and ^#^p<0.05 *versus* the leptin-treated rats). The protein bands are the same size as bovine serum albumin (BSA, 69 kDa), which suggests albumin excretion, namely albuminuria.

### Intrarenal mRNA expression

**Fig 8:**
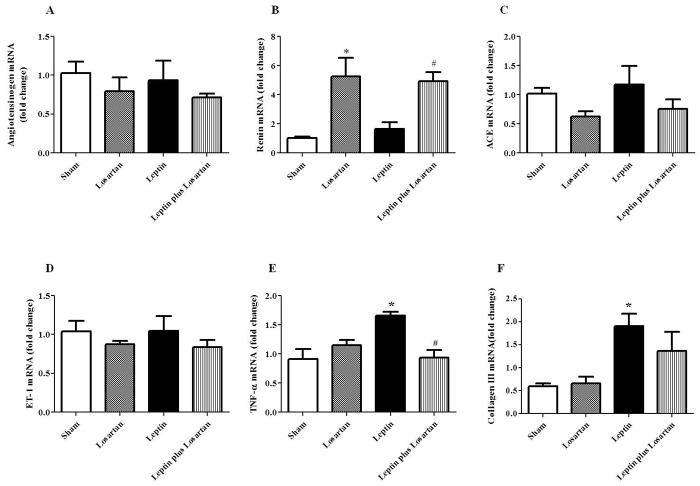
Quantitative PCR. Quantitative PCR analyses in the renal tissue of rats treated for 28 days (in fold change): angiotensinogen (A), renin (B), ACE (C), ET-1 (D), TNF-α (E) and collagen type III (F) from sham (n = 6), losartan (n = 6), leptin (n = 8) and leptin plus losartan (n = 6) treated rats. *p<0.05 *versus* the sham and ^#^p<0.05 *versus* the leptin-treated rats.

The expression of the mRNA of leptin receptors (Ob-Ra and Ob-Rb) was not changed during leptin and/or losartan treatment (Table 4). Many studies demonstrated that in some hypertensive models, there is activation of the intrarenal RAS, associated with increased intrarenal synthesis of angiotensinogen, angiotensin I, angiotensin converting enzyme (ACE) and Ang II [40–42]. Once our results showed an important contribution of the Ang II/AT_1_ receptor on leptin’s effects, we next investigated whether leptin treatment for 28 days enhances the expression of intrarenal RAS components. Our results showed no differences in angiotensinogen and ACE mRNA expression in renal tissue from rats treated with leptin for 28 days. The expression of renin mRNA was increased in both the losartan and the leptin plus losartan groups compared with the sham rats (Table 4 and Fig. 8A-C). The expression of ET-1 mRNA was also not different among the studied groups (Table 4 and Fig. 8D). The results in the rats treated with leptin showed a significant increase in the expression of the inflammatory cytokine TNF-α, which was reduced in the leptin plus losartan group (Table 4 and Fig. 8E). The expression of the fibrotic protein collagen III also increased in the leptin-treated rats, however it was not normalized by co-treatment with losartan (Table 4 and Fig. 8F).

**Table 4:** Quantitative PCR of RAS components in renal tissue of rats treated for 28 days.

	Sham n = 6	Losartan n = 6	Leptin n = 8	Leptin plus Losartan n = 6
**Ob-Ra mRNA (fold change)**	1.06 ± 0.18	1.05 ± 0.05	0.95 ± 0.06	0.98 ± 0.23
**Ob-Rb mRNA (fold change)**	1.09 ± 0.26	1.27 ± 0.12	1.36 ± 0.06	1.60 ± 0.16
**Agt mRNA (fold change)**	1.03 ± 0.15	0.79 ± 0.18	0.93 ± 0.25	0.72 ± 0.05
**Renin mRNA (fold change)**	1.01 ± 0.11	5.24 ± 1.28*	1.66 ± 0.45	4.94 ± 0.59^#^
**ACE mRNA (fold change)**	1.01 ± 0.10	0.62 ± 0.09	1.17 ± 0.32	0.75 ± 0.17
**ET-1 mRNA (fold change)**	1.04 ± 0.13	0.87 ± 0.04	1.04 ± 0.19	0.83 ± 0.09
**TNF-α mRNA (fold change)**	0.91 ± 0.17	1.15 ± 0.09	1.65 ± 0.06*	0.94 ± 0.13^#^
**Col III mRNA (fold change)**	0.59 ± 0.06	0.65 ± 0.15	1.90 ± 0.27*	1.36 ± 0.42

Agt (Angiotensinogen); ACE (Angiotensin Converting Enzyme); TNF-α (Tumor Necrosis Factor), TGF-β (Transforming Growth Factor); Col III (Collagen type III); ET-1 (Endothelin 1); Ob-Ra (Short leptin receptor isoform); Ob-Rb (Long leptin receptor isoform).

*p< 0.05 *versus* Sham;

^#^p< 0.05 *versus* Leptin.

## Discussion

Although many studies have accumulated evidences that leptin is a multifunctional hormone with actions extending beyond the regulation of the appetite and body weight, we did not observe changes in food intake of rats treated with leptin for 7 or 28 days, in the present study. These findings are in agreement with other studies [17, 28, 43]. The decrease in body weight gain observed in the 7-day group may be related to altered energy expenditure and an enhanced metabolic rate. The absence of differences in body weight gain in rats treated for 28 days suggest a resistance to the anorexic effects of leptin. In part, our data are consistent with the findings of Gunduz *et al*. [18], which demonstrated that rats treated with leptin for 28 days had the same food intake as the sham rats, but had less body weight gain. However, the leptin dose used in this study was much higher than in our study, suggesting a dose-response effect of leptin on body weight gain.

Our results also demonstrated that leptin treatment for 7 and 28 days induced, as expected, a significant increase in the plasma leptin levels. Surprisingly, the rats treated with leptin plus losartan showed lower plasma leptin levels, which suggests an interaction between leptin and the Ang II/AT_1_ receptor in controlling this parameter. In addition, Skurk *et al*. [44] demonstrated that Ang II stimulates leptin secretion from adipocytes, and this process involves the AT_1_ receptor. Because we blocked the binding of Ang II to AT_1_ receptors with losartan, less leptin was released from adipocytes. Despite these interactions between leptin and the Ang II/AT_1_ receptor, we did not observe changes to leptin receptor (the short isoform Ob-Ra and the long isoform Ob-Rb) expression in the renal tissue, of any group.

In obese animals, the development of hypertension is associated with high leptin levels [20, 45]. In fact, many studies have demonstrated that leptin induces an elevation of the SBP [14, 16, 28, 29, 46–49]. Our results are in agreement with these studies because leptin treatment induced a progressive increase in the SBP, achieving a pre-hypertensive state. In both treatment periods (7 and 28 days), the increase in the final SBP induced by leptin was prevented by losartan, suggesting a crosstalk between leptin and the Ang II/AT_1_ receptor to induce the increase in the SBP. Bornstein and Torpy [50] demonstrated that rats treated with leptin (0.12 mg/kg) for 7 days showed increased plasma renin activity; however, the effects of leptin on Ang II were not studied. Our results showed increased Ang II plasma levels. Thus, to our knowledge, this is the first study to demonstrate changes in the SBP as a function of hyperleptinemia and associated with increased AngII/AT_1_ receptor. In the present study, the SBP did not change in the losartan-treated rats, which is in accordance with other studies [51, 52] and unpublished data from our group.

The effect of leptin on renal hemodynamics is not completely understood. Beltowski *et al*. [28, 49] and Gunduz *et al*. [18] showed no differences in the GFR and renal blood flow (RBF) in rats chronically treated with leptin for 7 and 28 days, respectively. However, Di Bona [53] suggested that hyperleptinemia could induce a reduction in the RPF, renal vasoconstriction, enhanced renin secretion and *de novo* Ang II synthesis. Our results are in accordance with Di Bona′s findings because in rats treated with leptin for 7 days, we observed a slight decrease (23%) in the RPF, and this decrease was progressive and achieved statistical significance in rats treated for 28 days. In both treatment periods, the RPF was normalized in the leptin plus losartan group, indicating that leptin acting on the sympathetic nervous system can induce vasoconstriction in the renal arterioles and activate the RAS. The discrepancy between our results and Gunduz`s findings can be justified by the different methods used. Gunduz *et al*. [18] evaluated the RBF by an indirect method, using a laser module system, whereas in our study, the RPF was analyzed through PAH clearance.

In the present study, leptin treatment for 7 or 28 days did not result in GFR changes. However, although not significantly different, the alterations of the GFR and RPF observed in the present study are physiologically relevant because the FF was significantly higher in rats treated with leptin for 7 and 28 days. Only the leptin plus losartan rats treated for 7 days displayed a completely normalized FF, suggesting that in the 28-day group, not only the Ang II/AT_1_ receptor, but also other factors such as ET-1 and NO participates in the FF enhancement. ET-1 is the main peptide of the endothelin family, which includes ET-2 and ET-3. ET-1 binds to two different receptors: ET_A,_ which mediates vasoconstriction and ET_B_, which mediates vasodilation and inflammatory processes [54]. Furthermore, in many diseases, the synthesis and activity of ET-1 is increased in the kidneys [55]. Gunduz *et al*. [18], studying hyperleptinemic rats, demonstrated an increase in the plasma ET-1 levels, which were normalized by co-treatment with losartan. In the present study, we did not observe changes in plasma ET-1 levels. However, of note, in the study performed by Gunduz et al., the leptin dose was much higher compared with the doses used by us. Furthermore, Beltowski et al. [17] demonstrated that leptin treatment induces renal oxidative stress and decreases NO availability. The NO deficiency contributes to the intrarenal resistance and induces hemodynamic changes, by modulating the FF.

The urinary flow rate increased in the leptin-treated rats for 7 days and was normalized in the leptin plus losartan group. In contrast, the rats treated for 28 days exhibited no differences in the urinary flow rate. Controversy exists regarding Na^+^ regulation with chronic leptin infusion. Kuo *et al*. (2001) [15] did not observe any differences in Na^+^ excretion, whereas Beltowski *et al*. (2004) [16] showed Na^+^ retention and Gunduz *et al*. (2005) [18] demonstrated a natriuretic effect. Villarreal *et al*. [56] suggested that the increase in the fractional excretion of Na^+^ without changes in the GFR could be due to a tubular mechanism. In fact, the identification of the long leptin receptor (Ob-Rb) in the renal medulla, primarily in the medullary collecting ducts, indicates that this segment is a possible target for leptin’s direct action [11, 57]. Our results showed a natriuretic effect with leptin treatment for 7 days, which was normalized in the leptin plus losartan group and is in concordance with the observed enhanced urinary flow rate (diuresis). Our results also indicated that the natriuretic effect might be due to tubular mechanisms because the Na^+^ filtration load and Na^+^ plasma levels are similar in the groups studied. However, the natriuretic effect was absent in the 28-day group. This indicates that the enhanced Na^+^ excretion in the 7-day group could be an effort to counterbalance the increase in the SBP, but this effect is blunted in the 28-day group. In fact, both the increases in renal sympathetic nerve activity and the activation of the RAS contribute to the modulation of the pressure natriuresis mechanism and impairs the ability of the kidneys to maintain blood pressure and sodium homeostasis [58]. The increase in the plasma Ang II levels observed in the 28-day leptin-treated rats further support these results. Considering that short leptin receptors (Ob-Ra) are expressed in the glomerulus [9], that long leptin receptors (Ob-Rb) are expressed in the medulla [11], and that we did not observe differences in leptin receptors mRNA expression after chronic treatment, we next investigated whether leptin treatment induces renal morphological changes. The peptide induced a significant increase in the glomerular area, namely glomerular hypertrophy, which worsened from 7 to 28 days of treatment. Treatment with leptin plus losartan decreased the hypertrophy, suggesting that Ang II via the AT_1_ receptor is at least partially responsible for this effect. Glomerular hypertrophy is considered a relevant event in the progression of glomerular injury [59]. The rats treated with leptin for 7 and 28 days exhibited enhanced desmin staining, which is an important marker of glomerular lesion and is associated with the observed hypertrophy. In normal rats, desmin is expressed mainly in mesangial cells. Desmin expression in podocytes occurs after injury; therefore, desmin staining can be used as a reliable marker of podocyte damage [60]. The albuminuria observed in the rats treated with leptin further confirms the leptin’s effects on glomerular injury.

The histological analysis also demonstrated that leptin treatment for 28 days induced interstitial damage, observed as an increase in the fractional interstitial area, which was normalized in the leptin plus losartan group. Moreover, the rats treated with leptin for 28 days showed a significant infiltration of macrophages in the renal tissue, which was confirmed by ED-1 staining, that indicates a local inflammatory process and corroborates with the interstitial damage. The infiltration of ED-1 positive cells was reduced in the leptin plus losartan group, which indicates the protective effect of the antagonism of the AT_1_ receptor.

The positive effects of leptin on the mRNA expression of fibrotic and inflammatory components are in agreement with the results presented so far. The increase in collagen III expression is associated with glomerular hypertrophy, and the increase in TNF-α is highly associated with inflammation of the renal tissue. TNF-α binding to TNF receptor type 1 appears to potentiate angiotensin II-induced hypertension by suppressing renal nitric oxide production [61] and also appears to induce renal vasoconstriction [62]. Furthermore, Loffreda et al. [63] demonstrated that leptin up-regulates inflammatory responses, activates macrophages and induces the expression of pro-inflammatory cytokines, such as TNF-α.

Because the effects of leptin on the SBP, renal function and morphology were more pronounced in the rats treated for 28 days, we next investigated the effect of leptin on mRNA expression of intrarenal RAS components. Our results did not reveal changes in the expression of these components as has been observed in other hypertensive models. The enhanced renin expression in the losartan and leptin plus losartan groups, results from the blockade of negative feedback on the synthesis and secretion of renin by juxtaglomerular cells. Ang II, via the AT_1_ receptors, classically inhibits renin secretion. Thus, the blockade of Ang II action by losartan induces renin secretion.

Collectively, the results presented here demonstrated that the crosstalk between leptin and systemic RAS induced a progressive increase in the SBP and activated systemic RAS, thereby increasing plasma Ang II levels. In turn, Ang II acting via AT_1_ receptor induced progressive glomerular injury, renal inflammation and consequently, renal dysfunction. These findings are relevant to understand how hyperleptinemia contributes to the progressive alterations in renal function and morphology and the development of hypertension in this experimental model.
